# Well being of obstetric patients on minimal blood transfusions (WOMB trial)

**DOI:** 10.1186/1471-2393-10-83

**Published:** 2010-12-16

**Authors:** Babette W Prick, Eric AP Steegers, AJ Gerard Jansen, Wim CJ Hop, Marie-Louise Essink-Bot, Nina CJ Peters, Carin A Uyl-de Groot, Dimitri NM Papatsonis, Bettina MC Akerboom, Godfried CH Metz, Henk A Bremer, Aren J van Loon, Rob H Stigter, Joris AM van der Post, Marcel van Alphen, Martina Porath, Robbert JP Rijnders, Marc EA Spaanderman, Daniela H Schippers, Kitty WM Bloemenkamp, Kim E Boers, Hubertina CJ Scheepers, Frans JME Roumen, Anneke Kwee, Nico WE Schuitemaker, Ben Willem J Mol, Dick J van Rhenen, Johannes J Duvekot

**Affiliations:** 1 Department of Obstetrics and Gynaecology, Erasmus Medical Centre, Rotterdam, the Netherlands; 2 Department of Internal Medicine, Maasstad Hospital, Rotterdam, the Netherlands; 3 Department Biostatistics, Erasmus Medical Centre, Rotterdam, the Netherlands; 4 Department of Public Health, Academic Medical Centre, Amsterdam, the Netherlands; 5 Institute for Medical Technology Assessment, Erasmus Medical Centre, Rotterdam, the Netherlands; 6 Department of Obstetrics and Gynaecology, Amphia Hospital, Breda, the Netherlands; 7 Department of Obstetrics and Gynaecology, Albert Schweitzer Hospital, Dordrecht, the Netherlands; 8 Department of Obstetrics and Gynaecology, Ikazia Hospital, Rotterdam, the Netherlands; 9 Department of Obstetrics and Gynaecology, Reinier de Graaf Gasthuis, Delft, the Netherlands; 10 Department of Obstetrics and Gynaecology, Martini Hospital, Groningen, the Netherlands; 11 Department of Obstetrics and Gynaecology, Deventer Hospital, Deventer, the Netherlands; 12 Department of Obstetrics and Gynaecology, Academic Medical Centre, Amsterdam, the Netherlands; 13 Department of Obstetrics and Gynaecology, Flevo Hospital, Almere, the Netherlands; 14 Department of Obstetrics and Gynaecology, Maxima Medical Centre, Veldhoven, the Netherlands; 15 Department of Obstetrics and Gynaecology, Jeroen Bosch Hospital, 's-Hertogenbosch, the Netherlands; 16 Department of Obstetrics and Gynaecology, Radboud University Nijmegen Medical Centre, Nijmegen, the Netherlands; 17 Department of Obstetrics and Gynaecology, Canisius Wilhelmina Hospital, Nijmegen, the Netherlands; 18 Department of Obstetrics and Gynaecology, Leiden University Medical Centre, Leiden, the Netherlands; 19 Department of Obstetrics and Gynaecology, Bronovo Hospital, The Hague, the Netherlands; 20 Department of Obstetrics and Gynaecology, Maastricht University Medical Centre, Maastricht, the Netherlands; 21 Department of Obstetrics and Gynaecology, Atrium Medical Centre, Heerlen, the Netherlands; 22 Department of Obstetrics and Gynaecology, University Medical Centre Utrecht, Utrecht, the Netherlands; 23 Department of Obstetrics and Gynaecology, Diakonessen Hospital, Utrecht, the Netherlands; 24 Sanquin Blood Supply Foundation, Rotterdam, the Netherlands

## Abstract

**Background:**

Primary postpartum haemorrhage is an obstetrical emergency often causing acute anaemia that may require immediate red blood cell (RBC) transfusion. This anaemia results in symptoms such as fatigue, which may have major impact on the health-related quality of life. RBC transfusion is generally thought to alleviate these undesirable effects although it may cause transfusion reactions. Moreover, the postpartum haemoglobin level seems to influence fatigue only for a short period of time. At present, there are no strict transfusion criteria for this specific indication, resulting in a wide variation in postpartum policy of RBC transfusion in the Netherlands.

**Methods/Design:**

The WOMB trial is a multicentre randomised non-inferiority trial. Women with acute anaemia due to postpartum haemorrhage, 12-24 hours after delivery and not initially treated with RBC transfusion, are eligible for randomisation. Patients with severe physical complaints are excluded. Patients are randomised for either RBC transfusion or expectant management. Health related quality of life (HRQoL) will be assessed at inclusion, at three days and one, three and six weeks postpartum with three validated measures (Multi-dimensional Fatigue Inventory, ShortForm-36, EuroQol-5D). Primary outcome of the study is physical fatigue three days postpartum. Secondary outcome measures are general and mental fatigue scores and generic health related quality of life scores, the number of RBC transfusions, length of hospital stay, complications and health-care costs.

The primary analysis will be by intention-to-treat. The various longitudinal scores will be evaluated using Repeated Measurements ANOVA. A costs benefit analysis will also be performed. The power calculation is based on the exclusion of a difference in means of 1.3 points or greater in favour of RBC transfusion arm regarding physical fatigue subscale. With missing data not exceeding 20%, 250 patients per arm have to be randomised (one-sided alpha = 0.025, power = 80%).

**Discussion:**

This study will provide evidence for a guideline regarding RBC transfusion in the postpartum patient suffering from acute anaemia. Equivalence in fatigue score, remaining HRQoL scores and physical complications between both groups is assumed, in which case an expectant management would be preferred to minimise transfusion reactions and costs.

**Trial registration:**

ClinicalTrials.gov NCT00335023, Nederlands Trial Register NTR335

## Background

One of the most frequent complications of delivery is primary postpartum haemorrhage (PPH), defined as blood loss greater than or equal to 500 ml within 24 hours after birth and severe PPH as blood loss greater than or equal to 1000 ml within 24 hours [1]. Between four and five percent of all vaginal deliveries are complicated by severe PPH [2]. Blood loss during caesarean section is usually 50-100% more than during vaginal delivery [3]. An important clinical effect of obstetric haemorrhage is the development of acute anaemia, with fatigue as an important symptom. This fatigue is not an isolated physical symptom but rather involves lethargy, decreased mental alertness, physical weakness and poor concentration [4]. It is especially important as it affects women who just have delivered, who have to care for and feed a newborn baby and who are usually full of emotions.

RBC transfusion is one of the few treatments that adequately restore tissue oxygenation when oxygen demand exceeds supply. Apart from the life-saving restoration of the initial hemodynamic instability, RBC transfusion is also used to alleviate the side-effects of acute anaemia, including the fatigue mentioned above. This treatment is applied frequently.  Previously, a significant correlation between Hb level postpartum and fatigue scores was found in postpartum patients using the HRQoL measure Multidimensional Fatigue Inventory (MFI), but this correlation was already lost one week after delivery [5]. Thus, postpartum haemoglobin (Hb) level seems to influence fatigue for only a short period of time. Other arguments against RBC transfusion are that RBC transfusion is costly and may cause formation of RBC alloantibodies and other direct and indirect transfusion complications [6].

Although the respiratory function of blood has been studied intensively, the trigger for RBC transfusion remains controversial, and doctors still rely primarily on clinical experience. At present, criteria for transfusion are not based on alternative criteria, such as HRQoL scores.  Worldwide there is no consensus on the optimal transfusion trigger [6-9]. A questionnaire study in 2003 on the subject of RBC transfusion practice postpartum in the Netherlands also showed large differences between hospitals [10]. Based on Hb levels, the transfusion triggers varied between 3.0 and 5.5 mmol/L (4.8 and 8.9 g/dL). Clinical symptoms were used as more or less subjective additional criteria.

The official Dutch guideline for RBC transfusion is the "4-5-6 flexinorm" for patients with acute or chronic anaemia [9]. This rule states that ASA I patients should have RBC transfusion when their Hb is below 4 mmol/L (6.4 g/dL), ASA II when their Hb is below 5 mmol/L (8.1 g/dL) and ASA III and IV patients when their Hb is below 6 mmol/L (9.7 g/dL). However, during pregnancy the circulating blood volume increases by 100 mL/kg to a total blood volume of six to seven liters. The several blood components contribute differently to this increase: plasma increases with 40% whereas red blood cell volume increases with 15-20%. Consequently Hb level decreases with a maximum of approximately 10%. This natural process of hemodilution improves the placental circulation. As a result of these changes, it is doubtful whether the Hb triggers for RBC transfusion, as described above, can be used for patients with postpartum haemorrhage.

In the Netherlands all women with PPH will be treated by a gynaecologist in a clinical setting. Although almost one third of Dutch women deliver at home with supervision by a midwife, women that experience excessive blood loss during or immediately after delivery will be transferred to a hospital for further treatment by a gynaecologist. Most Dutch women that have a hospital delivery, leave hospital a couple of hours after delivery since there is an adequately organised domiciliary postnatal care service. The necessity of RBC transfusion will prolong their stay in hospital.

In the Netherlands, blood products are produced by one blood banking organisation. The present study is performed in cooperation with Sanquin, the Dutch blood bank organisation, which initiated and funded the costs of the first part of this study.

Acute anaemia postpartum, with fatigue as important symptom, is a clinical problem with wide practice variation in treatment regarding RBC transfusion. There are several arguments against RBC transfusion and the postpartum Hb level seems to influence fatigue for only a short period of time. We therefore aim to show non-inferiority of expectant management versus a transfusion policy regarding physical fatigue, three days after delivery, in postpartum women with an acute anaemia following PPH. The development of a guideline based on clinical parameters, laboratory determinations and HRQoL measures may lead to a more adequate use of RBC transfusions in the postpartum period and possibly reduces costs due to lower frequency of transfusions and hospitalization duration.

## Methods/design

### Aims

The aim of this study is to solve the dilemma for the obstetrician regarding the optimal treatment of women with acute anaemia postpartum. For that reason we determine whether women with acute anaemia postpartum benefit from RBC transfusion.

Our hypothesis is that there is no important difference with regard to physical fatigue, as well as other HRQoL scores and physical complications between RBC transfusion and expectant management as treatment for women with acute postpartum anaemia in the absence of (severe) physical complaints.

If this hypothesis is true, an expectant management is to be preferred to minimise transfusion related reactions and costs.

### Study design

The study is a multicentre randomised non-inferiority trial in women with an acute postpartum anaemia. It is an open label study. This trial is embedded in the Dutch Obstetric Consortium, a collaboration of hospitals in the Netherlands [11]. Approximately 40 hospitals, including university hospitals, teaching hospitals and non-teaching hospitals will participate in this trial.

### Participants/eligibility criteria

Women, older than 18 years of age, who deliver in hospital or are transferred after home delivery because of PPH, are eligible. Patients will be included with an Hb between 3.0 and 5.0 mmol/L (4.8 and 8.1 g/dL), determined 12 to 24 hours after vaginal delivery or caesarean section, and a decrease in Hb of at least 1.2 mmol/L (1.9 g/dL) and/or a total peripartum blood loss of at least 1000 mL. The initial Hb value will be determined when the patient is admitted during the first stage of labour at the labour ward. In other instances, when an initial Hb is absent, inclusion is purely based on the total amount of blood loss. Exclusion criteria include severe (anaemic) physical complaints, previous RBC transfusion directly after delivery, severe pre-eclampsia, severely active infectious disease, congenital haemolytic disease, severe compromised immunological status, malignancy, severe co-morbidity (ASA II/III), peripartum death or critical condition of the newborn. Severe (anaemic) physical complaints were defined as fatigue, headache, dizziness, confusion, dyspnoea, syncope, orthostatic complaints, tachycardia (> 100 bpm), angina pectoris and/or transient ischemic attacks (TIA). Finally, good working knowledge of the Dutch language is required.

### Procedures, recruitment, randomisation and collection of baseline data

Eligible patients receive participant information. After written consent, participants are randomised by means of a web-based application [12]. Randomisation will be blocked in a 1:1 ratio for RBC transfusion or expectant management. Stratification will be applied for mode of delivery and participating hospital.

All data will be collected, coded and processed with adequate precautions to ensure patient confidentiality.

### Intervention and control

RBC transfusion will be compared with expectant management.

In patients allocated to RBC transfusion, at least one unit of packed cells will be given. The desired Hb level after transfusion is 5.5 mmol/L (8.9 g/dL). The units of blood will be matched, pre-treated and tested according to the Dutch guidelines for RBC transfusion. Before and 15-90 minutes after transfusion maternal body temperature, blood pressure and heart rate will be checked and a blood sample is taken to determine Hb, haematocrit, platelet and white blood count.

In the group allocated to expectant management, i.e. no RBC transfusion, it is allowed to give a patient 'rescue' RBC transfusion if this is clinically indicated. The indications to give 'rescue' RBC transfusion are secondary PPH, resulting in hemodynamic instability or an Hb value < 3.0 mmol/L (4.8 g/dL), serious physical complaints or other serious general complications. Possible serious physical complaints are: dyspnoea, syncope, orthostatic complaints, tachycardia (> 100 bpm), myocardial ischemia or transient ischemic attacks (TIA).

Additional use of medication to treat anaemia, like oral and parenteral iron medication and other types of medication, is allowed in both groups and may be prescribed according to local protocol. Type and duration of the use of this medication will be recorded.

### Outcome

Primary outcome is physical fatigue on day three postpartum, scored with a dimension of the HRQoL measure MFI. We aim to show non-inferiority of the expectant management arm and therefore this primary assessment day was chosen: if there would be any difference between randomized groups, this difference is expected to be the largest three days postpartum [5]. In total three measures of HRQoL will be scored. Secondary outcomes will be the remaining HRQoL measures and the number of RBC transfusions. In addition an anaemia related symptoms list will be filled in before randomisation and at six weeks postpartum.

Medical process parameters to be evaluated include the increase of Hb level after RBC transfusion and acute transfusion complications. Moreover, we will compare the length of hospital stay and serious physical complications during the first six weeks postpartum (infections, thromboembolic events, hemodynamic events, cardiac events, neurologic events, secondary HPP, obstetric interventions, 'rescue' RBC transfusion). A costs benefit analysis will also be performed (see 'Economic Evaluation').

### HRQoL questionnaires

HRQoL will be scored with three measures: the generic measures Short Form-36 (SF-36) and EuroQol-5 D (EQ-5D) and the fatigue specific measure MFI.

The SF-36 is a multi-purpose, short-form health survey with only 36 questions. It consists of 36 items, organized into eight scales: Physical Functioning, Role-Physical, Bodily Pain, General Health, Vitality, Social Functioning, Role-Emotional, and Mental Health. The number of response choices per item ranges from two to six. The SF-36 yields an eight-dimensional profile, with each scale having a range from 0 to 100 (100 = optimal). The SF-36 furthermore provides a physical summary score and a mental summary score. The SF-36 has proven useful in surveys of general and specific populations, comparing the relative burden of diseases, and in differentiating the health benefits produced by a wide range of different treatments [13,14].

The EQ-5 D is a standardised measure of health status and provides a simple, generic measure of health for clinical and economic appraisal. It consists of five items (Mobility, Self-Care, Usual Activities, Pain/Discomfort, and Anxiety/Depression), each following the general form: 1 = *no problems*, 2 = *some problems*, 3 = *extreme problems*. The sixth item is a global evaluation of own health on a visual analogue scale (EQ-VAS) with a range from 0 (*worst imaginable health state*) to 100 (*best imaginable health state*). Applicable to a wide range of health conditions and treatments, it provides a simple descriptive profile and a single index value for health status that can be used in the clinical and economic evaluation of health care as well as in population health surveys [15].

The MFI is a 20-item self-report instrument designed to measure fatigue. It covers the following dimensions: General Fatigue, Physical Fatigue, Mental Fatigue, Reduced Motivation and Reduced Activity. Each dimension is covered by a five-item scale. The number of response choices per item is four. Each scale has ranges from 4 to 20 (4 = optimal). The MFI was developed as a tool to assess fatigue in a comprehensive way, with a special interest in fatigue as experienced by patients and provides information on the nature of the experience, and its intensity [16].

The international standard HRQoL measures MFI, EQ-5 D, and SF-36 have been evaluated in a pilot study; feasibility, reliability, and validity of these measures in a clinical obstetric setting were established [5].

### Follow-up

The follow-up period consists of six weeks. At fixed moments during this period (at inclusion, three days, one, three and six weeks) HRQoL questionnaires will completed. The EQ-5 D and MFI will be filled in at all these fixed moments while the SF-36 will be used one, three and six weeks postpartum.

Details of the delivery, RBC transfusion (when given) and follow-up are recorded in the CRF. During the study period all transfusion complications will be recorded. An anaemia related symptoms list is filled in before randomisation and at six weeks postpartum. Hb values will be determined at inclusion and six weeks postpartum. Six weeks postpartum patients visit the outpatient clinic.

In case of severe adverse events (SAE) during follow-up, a SAE form will be downloaded from the website and completed. SAE forms will be judged and based on this conclusion the study will be discontinued if participation in the study is considered irresponsible.

Patients that withhold informed consent for randomisation will be asked to complete the questionnaires, and case record forms (CRF) will be recorded in a similar way as patients that were randomised.

### Statistics

#### Sample size

The HRQoL assessments were tested in postpartum women in a pilot study [5]. For the comparison between RBC transfusion and an expectant management a sample size of 400 patients is planned (200 patients in the RBC transfusion group and 200 patients in the expectant management group).  Power calculations showed that with these numbers a difference of 1.3 points or greater in favour of the RBC arm regarding the physical fatigue subscale on day three, the primary outcome, can be excluded with a power of 80% at one-sided alpha of 0.025 assuming that there is no difference on this day [5]. In view of the scale of physical fatigue (ranging from 4 to 20) we think that such a difference is not clinically relevant. Since missing data are not expected to exceed 20%, a total of 500 patients will be included.

#### Data analysis

Data will be analysed according to the intention to treat principle. The primary outcome, the MFI physical fatigue score at three days postpartum, will be compared between groups using Repeated Measurement ANOVA. This analysis allows for occasional missing values and will be done using an unstructured covariance matrix, while taking account of baseline value (at t = 0) and mode of delivery as covariates. If the two-sided 95% confidence interval for the adjusted difference of means at day three excludes a difference of 1.3 points or greater in favour of the RBC arm, non-inferiority of the expectant policy is considered to be shown [17]. A per-protocol analysis, including only patients without severe protocol violations, will also be performed.

The mean profiles along time of other MFI subscales, SF-36 and EQ-5 D will be compared similarly to the primary outcome measure. The other secondary outcome measures will be assessed by calculating rates in groups, risk differences and 95% confidence interval.

#### Economic evaluation

The aim of the economic evaluation is to compare the costs and health effects of RBC transfusion versus expectant management in women with acute anaemia after PPH. As the clinical study is designed as an equivalence study, the primary economic evaluation is a cost-minimisation analysis. If RBC transfusion is shown to improve the quality of life, the economic analysis will be a cost-effectiveness analysis (time horizon: six weeks postpartum). The analysis will be performed from a hospital perspective. We will calculate direct costs (days in hospital, number of RBC transfusions and costs of complications). Real medical costs will be calculated by multiplying the volumes of health care use with the corresponding cost prices. Unit costs of health care consumption will be estimated according to national guidelines. Data on the volume of care will be available from hospital information systems in the participating hospitals.

Univariate analyses will be used for the analysis of economic outcome data. We will use non-parametric methods to test for differences between treatment groups.

### Ethical considerations

This study has been approved by the ethics committee of the Erasmus Medical Centre Rotterdam (Ref. No. MEC-2003-247) and by the management of all participating hospitals.

## Discussion

Yearly, almost 200.000 women deliver in the Netherlands [18]. The number of women that receive one or more RBC transfusions postpartum is estimated less than 1% after vaginal delivery and between 1% and 7% following caesarean section [3]. The present study may lead to transfusion guidelines that are not only based on Hb levels and/or clinical symptoms but also on HRQoL measures. This may lead to a more judicious use of RBC transfusions.

To our knowledge, this is the first study investigating worldwide the effect of RBC transfusion on postpartum HRQoL in women with acute anaemia due to PPH. A pilot study investigating the relation between postpartum Hb levels and HRQoL measures during the first six weeks postpartum, which is discussed below, was published by our group in 2007 [19].

Several national health organisations have tried to define transfusion triggers [20-24]. General consensus of these guidelines is that RBC transfusion above 6 mmol/L (9.7 g/dL) is not very useful, RBC transfusion below 4 mmol/L (6.4 g/dL) is probably useful, but with an Hb value between 4 and 6 mmol/L individual characteristics have to be taken in consideration for the decision to give RBC transfusion. In the Netherlands, the Dutch "4-5-6 flexinorm" is empirically based and is intended for use in the treatment of acute and chronic cases of anaemia [9]. However, considering the physiologic hemodynamic changes during pregnancy and the postpartum period in obstetric patients, the guideline might not be applicable for this specific patient group.

The hypothesis of the present study is that HRQoL is not or only to a small extent influenced by treatment with RBC transfusion in this group of patients. The results of our previous pilot study showed no correlation between postpartum Hb levels and HRQoL after the first week postpartum. The health effects of this study will be the direct reduction of RBC transfusions, which will result in a decrease of RBC transfusion-related complications (formation of RBC alloantibodies, transfusion complications and infectious complications) and days in hospital. Another possible finding of this study may be the definition of the desired Hb value after RBC transfusion.

## Abbreviations

Bpm: beats per minute; CRF: Case Record Form; EQ-5D: EuroQol-5 D; Hb: Haemoglobin; HRQoL: Health Related Quality of Life; MFI: Multidimensional Fatigue Inventory; PPH: Primary Postpartum Haemorrhage; RBC: Red Blood Cell; SAE: Severe Adverse Event; SF-36: Short Form-36.

## Competing interests

The authors declare that they have no competing interests.

## Authors' contributions

AJGJ, WCJH, MLEB, CAUG, BWM, DJR and JJD were involved in conception and design of the study. DNMP, BMCA, GCHM, HAB, AJL, RHS, JAMP, MA, MP, RJPR, MEAS, DHS, KWMB, KEB, HCJS, FJMWR, AK, NWES have made substantial contributions to acquisition of data. BWP, NCJP and JJD drafted the manuscript. All authors have read and given final approval of the final manuscript.

## Pre-publication history

The pre-publication history for this paper can be accessed here:

http://www.biomedcentral.com/1471-2393/10/83/prepub
